# Casual effects of type 1 diabetes mellitus on site-specific digestive cancers: a Mendelian randomisation analysis

**DOI:** 10.3389/fendo.2024.1407329

**Published:** 2024-09-05

**Authors:** Jinli Zhao, Wenjin Li, Libo Chen, Mingyong Li, Weiming Deng

**Affiliations:** ^1^ Department of Emergency Medicine, The First Affiliated Hospital, Hengyang Medical School, University of South China, Hengyang, China; ^2^ Department of Nutrition, The Second Affiliated Hospital, Hengyang Medical School, University of South China, Hengyang, China; ^3^ Department of Urology, The First Affiliated Hospital, Hengyang Medical School, University of South China, Hengyang, China

**Keywords:** diabetes mellitus, oesophageal cancer, stomach cancer, hepatocellular carcinoma, biliary tract cancer, pancreatic cancer, colorectal cancer, Mendelian randomisation

## Abstract

**Objective:**

Despite several observational studies attempting to investigate the potential association between type 1 diabetes mellitus (T1DM) and the risk of digestive cancers, the results remain controversial. The purpose of this study is to examine whether there is a causal relationship between T1DM and the risk of digestive cancers.

**Methods:**

We conducted a Mendelian randomisation (MR) study to systematically investigate the effect of T1DM on six most prevalent types of digestive cancers (oesophageal cancer, stomach cancer, hepatocellular carcinoma, biliary tract cancer, pancreatic cancer, and colorectal cancer). A total of 1,588,872 individuals were enrolled in this analysis, with 372,756 being the highest number for oesophageal cancer and 3,835 being the lowest for pancreatic cancer. Multiple MR methods were performed to evaluate the causal association of T1DM with the risk of six site-specific cancers using genome-wide association study summary data. Sensitivity analyses were also conducted to assess the robustness of the observed associations.

**Results:**

We selected 35 single nucleotide polymorphisms associated with T1DM as instrumental variables. Our findings indicate no significant effect of T1DM on the overall risk of oesophageal cancer (OR= 0.99992, 95% CI: 0.99979-1.00006, P= 0.2866), stomach cancer (OR=0.9298,95% CI: 0.92065-1.09466, P= 0.9298), hepatocellular carcinoma (OR= 0.99994,95% CI: 0.99987-1.00001, P= 0.1125), biliary tract cancer (OR=0.97348,95% CI: 0.8079-1.1729, P= 0.7775)), or pancreatic cancer (OR =1.01258, 95% CI: 0.96243-1.06533, P= 0.6294). However, we observed a causal association between T1DM and colorectal cancer (OR=1.000, 95% CI: 1.00045-1.0012, P<0.001), indicating that T1DM increases the risk of colorectal cancer. We also performed sensitivity analyses, which showed no heterogeneity or horizontal pleiotropy. For the reverse MR from T1DM to six digestive cancers, no significant causal relationships were identified.

**Conclusions:**

In this MR study with a large number of digestive cancer cases, we found no evidence to support the causal role of T1DM in the risk of oesophageal cancer, stomach cancer, hepatocellular carcinoma, biliary tract cancer, or pancreatic cancer. However, we found a causal positive association between T1DM and colorectal cancer. Further large-scale prospective studies are necessary to replicate our findings.

## 1 Introduction

The connection between diabetes and cancer has been a subject of scientific inquiry for over a century (1). Recent reports have indicated that patients diagnosed with diabetes mellitus are at a higher risk of developing cancer by 20-25% compared to those without diabetes (2). The susceptibility to various forms of cancer seems to be elevated in individuals with both Type 1 Diabetes Mellitus (T1DM) and Type 2 Diabetes Mellitus (T2DM) (3). In addition, numerous studies have shown that a significant percentage (estimated at 8% to 18%) of cancer patients also have diabetes (4) and an increased risk of cancer-related death (5). Cancer has been noted as the second leading cause of mortality among individuals with diabetes (6). This risk is particularly associated with T2DM, which is associated with an increased risk of certain site-specific cancers (7). Similarly, previous cohort studies have reported higher cancer standardised mortality in patients with T1DM (8). However, the association between T1DM and cancer remains unclear due to a limited number of studies T1DM. Furthermore, although hyperglycaemia is common in both types of diabetes mellitus, insulin resistance and hyperinsulinemia are more pronounced in T2DM than in T1DM (9). Additionally, T2DM is less exposed to exogenously administered insulin than T1DM. Therefore, findings on the association between T2DM and the risk of cancer cannot be directly applied to T1DM due to differences in age, obesity, and underlying mechanisms between the two patient groups. These findings highlight the need for further research to better understand the relationship between T1DM and cancer.

A multi-nation study has identified a strong link between T1DM and an increased risk of common cancers. The hazard ratio for overall cancer risk in T1DM patients was 1.15 (1.11, 1.19) for men and 1.17 (1.13, 1.22) for women, surpassing that of the general population. Both sexes with T1DM exhibited a notably higher incidence of solid malignant tumor (10, 11). Oesophageal, stomach, liver, biliary tract, pancreatic, and colorectal cancers, as most prevalent digestive cancers (12, 13), are a significant health concern and impose an increasing disease burden. The risk factors for digestive cancers have been widely investigated and are shared to a great degree (14). Despite the enormous efforts made to combat digestive cancers, there is still a long way to go to reduce the disease burden of digestive cancers. Proper management and early intervention at the onset of the condition can help improve the chances of treatment success and reduce the negative impact of digestive cancers (15). Therefore, it is crucial to identify modifiable protective factors that may help alleviate the burden of the disease (16). A better understanding of the risk factors associated with digestive cancers and the adoption of preventative measures may aid in reducing the incidence of this disease and offer a better prognosis for patients (17).

Mendelian randomisation (MR) analysis has emerged as a popular method for evaluating the potential relationship between exposure factors and outcomes (18). Unlike observational studies, MR analysis is not limited by confounding and reverse causality, as it relies on genetic variations associated with exposure factors to assess their association with outcomes. This is achieved through the use of single-nucleotide polymorphisms (SNPs) as instrumental variables (IVs) to determine disease incidence (19). High-throughput genome-wide association studies (GWASs) have enabled further investigation of causal effects (20). Despite the potential benefits of MR analysis, there is still a scarcity of studies examining the potential causal relationship between T1DM and digestive cancers. Therefore, the authors of this study conducted an MR analysis utilising high-throughput GWASs to assess the potential causal associations between T1DM and digestive cancers.

## 2 Materials and methods

### 2.1 Data sources

Summary statistics of T1DM and six Digestive system tumor from the largest available genome-wide association studies (GWAS) of European ancestry and FinnGen biobank were extracted for the primary MR analysis (21, 22). **Supplementary Table 1** presents a summary of various studies conducted on the association between different traits and cancer types in European populations. The studies include research on T1DM, oesophageal cancer, stomach cancer, hepatocellular carcinoma, biliary tract cancer, pancreatic cancer, and colorectal cancer. The sample sizes of these studies range from 3,835 to 377,673 individuals, and the number of cases and controls varies depending on the specific cancer type. Moreover, we assessed the causal effects, meticulously adjusting for a range of potential confounders to enhance the precision of our findings. We effectively controlled for variables such as obesity, body mass index, T2DM, high-density lipoprotein (HDL), low-density lipoprotein (LDL), triglycerides, and apolipoproteins (rs6679677, rs6909461, rs506770, rs9468618, rs689, rs10774624,rs10774624, rs8056814 and rs34536443 were eliminated). Furthermore, to delve deeper into the underlying mechanisms, we performed a mediation analysis aimed at uncovering whether these traits act as mediators in the causal relationship between T1DMand six distinct types of digestive cancers. These studies provide valuable insights into the potential genetic associations between different traits and cancer types, which could inform future research and public health efforts aimed at reducing the burden of digestive cancers. Since all the necessary data were publicly available online, no ethical approval or informed consent was required.

### 2.2 Selection of SNPs

This approach provides a rigorous and systematic method for selecting IVs in MR analysis, which is essential for ensuring the validity and reliability of the results. In this study, high-throughput GWASs were utilised to extract IVs for digestive cancer. The SNPs that reached a genome-wide significance level (P < 5 × 10^–8^) were selected as IVs (23). However, if fewer than five IVs were selected, the P value threshold for including SNPs as IVs was lowered to P < 1 × 10^-5^, which is a method that has been previously adopted in MR studies (24). SNPs within 10,000 kb of each other were then clumped, with a linkage disequilibrium threshold of R^2^ > 0.001 (25). The F-statistics of the IVs were estimated, which is an indicator of the ability of the IVs to predict the exposures. All exposures had F-statistics higher than 10, indicating that the selected IVs were strong predictors of the exposures.

### 2.3 Statistical analysis

The inverse-variance weighted (IVW) MR method was the primary method used in this study to ascertain the relationships between T1DM and different types of cancer risk. In addition to the IVW method, sensitivity analyses were conducted using the weighted median, MR-Egger, simple mode, and weighted mode test. The potential heterogeneity was estimated using Cochrane’s Q statistic, and the potential pleiotropy was assessed by the intercept of the MR-Egger test. Scatter plots were used to present the results of different MR methods. To assess the robustness of the results, a “leave-one-out” analysis was conducted to estimate the effect of SNPs after removing each SNP one by one. The causal effects of overall and site-specific cancer were represented using odds ratios (ORs) and 95% confidence intervals (CIs). The analyses were conducted using R software, with the “TwoSampleMR” R package employed for the analyses.

## 3 Results

A total of 35 SNPs associated with T1DM were chosen for analysis, as shown in **Supplementary Table 2**. For the reverse MR from T1DM to six digestive cancers, no significant causal relationships were identified.

### 3.1 Oesophageal cancer

**Figure 1:**
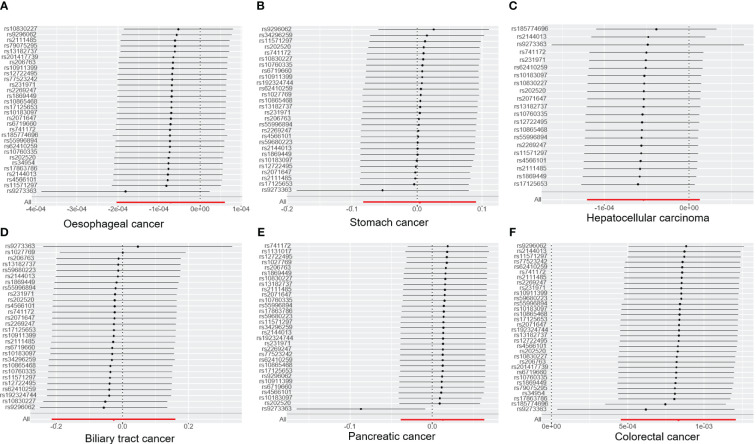
The forest plot illustrates the association between T1DM and digestive cancers, where each black dot represents an SNP. **(A)** oesophageal cancer. **(B)** stomach cancer. **(C)** hepatocellular carcinoma. **(D)** biliary tract cancer. **(E)** pancreatic cancer. **(F)** colorectal cancer. T1DM, type 1 diabetes mellitus; SNP, single-nucleotide polymorphism.

**Figure 2:**
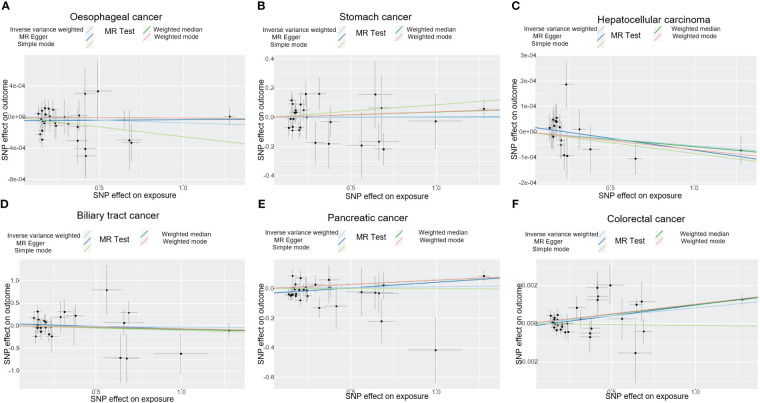
The plot displays the estimated causal effect of T1DM and the risk of digestive cancers based on various methods. The black dots in the graph signify SNPs with an estimated effect on T1DM and digestive cancers risk, while the slopes of the lines represent the causal-effect. **(A)** oesophageal cancer. **(B)** stomach cancer. **(C)** hepatocellular carcinoma. **(D)** biliary tract cancer. **(E)** pancreatic cancer. **(F)** colorectal cancer. T1DM, type 1 diabetes mellitus; SNP, single-nucleotide polymorphism.

**Figure 3:**
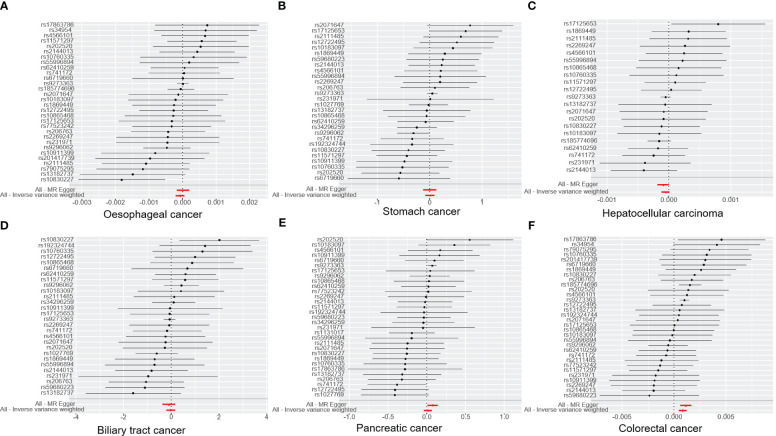
The graph illustrates the association of T1DM with digestive cancers risk and estimates and confidence intervals when a particular SNP is removed. **(A)** oesophageal cancer. **(B)** stomach cancer. **(C)** hepatocellular carcinoma. **(D)** biliary tract cancer. **(E)** pancreatic cancer. **(F)** colorectal cancer. T1DM, type 1 diabetes mellitus; SNP, single-nucleotide polymorphism.

**Figure 4:**
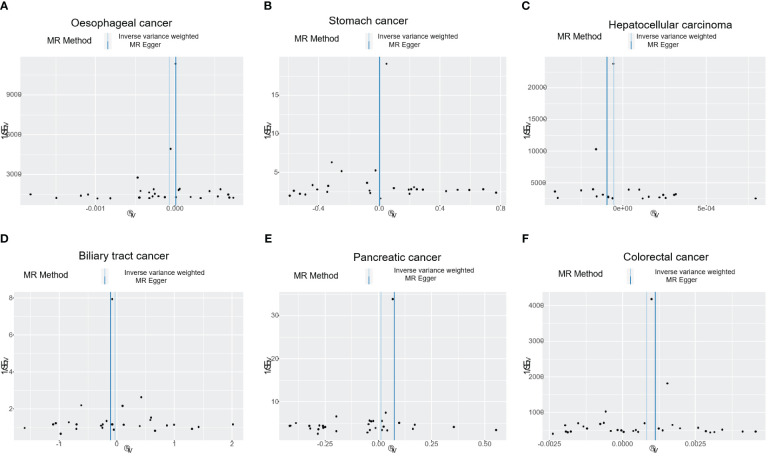
The funnel plot shows the correlation between T1DM and digestive cancers risk, with each black dot representing an SNP. **(A)** oesophageal cancer. **(B)** stomach cancer. **(C)** hepatocellular carcinoma. **(D)** biliary tract cancer. **(E)** pancreatic cancer. **(F)** colorectal cancer. T1DM, type 1 diabetes mellitus; SNP, single-nucleotide polymorphism.

The results of the IVW analysis indicated that there was no significant association between T1DM and oesophageal cancer (OR=0.99992, 95% CI: 0.99979-1.00006, P= 0.2866, **Table 1**), which was consistent with the results of the weighted median, MR-Egger, simple mode, and weighted mode analyses. The absence of directional pleiotropy was confirmed by the MR-Egger intercept test (P= 0.9037), and the heterogeneity did not reach statistical significance (P= 0.5832), as assessed by Cochran’s Q test. The forest and scatter plots, presented in **Figures 1A**, **2A**, respectively, further support the lack of association between T1DM and oesophageal cancer. The sensitivity analysis, shown in **Figure 3A**, revealed that the overall estimates were not significantly influenced by any individual SNP. Additionally, the funnel plot, displayed in **Figure 4A**, showed no evidence of horizontal pleiotropy.

**Table 1:** Mendelian randomisation estimates for the effects of genetically determined T1DM on six site-specific digestive cancers.

Site-specific digestive cancers	Method	SNPs	OR (95% CI)	P-value
Oesophageal cancer	Inverse variance weighted	30	1.00001 (0.99982-1.0002)	0.9037
	MR Egger	30	0.99999 (0.99982-1.00015)	0.9118
	Weighted median	30	0.99992 (0.99979-1.00006)	0.2866
	Simple mode	30	0.99974 (0.99929-1.00019)	0.2831
	Weighted mode	30	0.99998 (0.99983-1.00014)	0.8789
Stomach cancer	Inverse variance weighted	28	1.00102 (0.88308-1.13471)	0.9873
	MR Egger	28	1.03711 (0.93747-1.14733)	0.4795
	Weighted median	28	1.00389 (0.92065-1.09466)	0.9298
	Simple mode	28	1.0902 (0.79205-1.50059)	0.6005
	Weighted mode	28	1.03925 (0.93905-1.15014)	0.463
Hepatocellular carcinoma	Inverse variance weighted	21	0.9999 (0.99981-1)	0.0661
	MR Egger	21	0.99994 (0.99986-1.00002)	0.1624
	Weighted median	21	0.99994 (0.99987-1.00001)	0.1125
	Simple mode	21	0.99991 (0.9997-1.00012)	0.4476
	Weighted mode	21	0.99993 (0.99985-1)	0.0739
Biliary tract cancer	Inverse variance weighted	28	0.8993 (0.6898-1.1723)	0.4398
	MR Egger	28	0.9226 (0.7217-1.1793)	0.5200
	Weighted median	28	0.9735 (0.8079-1.1729)	0.7775
	Simple mode	28	0.9021 (0.4559-1.785)	0.7696
	Weighted mode	28	0.9342 (0.7446-1.172)	0.5613
Pancreatic cancer	Inverse variance weighted	31	1.07841 (1.0095-1.15203)	0.0328
	MR Egger	31	1.05868 (0.99971-1.12114)	0.0511
	Weighted median	31	1.01258 (0.96243-1.06533)	0.6294
	Simple mode	31	0.99823 (0.80038-1.245)	0.9876
	Weighted mode	31	1.05861 (1.00255-1.11781)	0.0489
Colorectal cancer	Inverse variance weighted	32	1.00112 (1.00058-1.00166)	0.0002
	MR Egger	32	1.00098 (1.00052-1.00144)	0
	Weighted median	32	1.00083 (1.00045-1.0012)	0
	Simple mode	32	0.9999 (0.99821-1.0016)	0.9151
	Weighted mode	32	1.00102 (1.00059-1.00145)	0

SNP, single-nucleotide polymorphism; OR, odds ratios; CI, confidence interval.

### 3.2 Stomach cancer

The results of our study consistently showed no causal associations between T1DM and stomach cancer, with an OR of 0.9298 (95% CI: 0.92065-1.09466, P= 0.9298), as presented in **Table 1**. The weighted median, MR-Egger, simple mode, and weighted mode analyses produced consistent estimates, and no evidence of directional pleiotropy was detected (P= 0.9873). Additionally, the heterogeneity was not statistically significant (P= 0.5832). The forest and scatter plots, displayed in **Figures 1B**, **2B**, respectively, further support the lack of association between T1DM and stomach cancer. The sensitivity analysis, presented in **Figure 3B**, revealed that no individual SNP caused the MR estimates to deviate. Furthermore, the funnel plot, illustrated in **Figure 4B**, showed no indication of horizontal pleiotropy.

### 3.3 Hepatocellular carcinoma

Our study consistently found no evidence of causal associations between T1DM and hepatocellular carcinoma, with an OR of 0.99994 (95% CI: 0.99987-1.00001, P= 0.1125), as presented in **Table 1**. The weighted median, MR-Egger, simple mode, and weighted mode analyses produced consistent estimates, and no evidence of directional pleiotropy was detected (P= 0.602). Additionally, the heterogeneity was not statistically significant (P= 0.7815). The forest and scatter plots, displayed in **Figures 1C**, **2C**, respectively, further support the lack of association between T1DM and hepatocellular carcinoma. The sensitivity analysis, presented in **Figure 3C**, revealed that no individual SNP caused the MR estimates to deviate. Furthermore, the funnel plot, illustrated in **Figure 4C**, showed no indication of horizontal pleiotropy.

### 3.4 Biliary tract cancer

Our findings consistently revealed no evidence of causal associations between T1DM and biliary tract cancer, with an OR of 0.97348 (95% CI: 0.8079-1.1729, P= 0.7775), as presented in **Table 1**. The weighted median, MR-Egger, simple mode, and weighted mode analyses produced consistent estimates, and no evidence of directional pleiotropy was detected (P= 0.2575). Additionally, the heterogeneity was not statistically significant (P= 0.7815). The forest and scatter plots, displayed in **Figures 1D**, **2D**, respectively, further support the lack of association between T1DM and biliary tract cancer. The sensitivity analysis, presented in **Figure 3D**, revealed that no individual SNP caused the MR estimates to deviate. Furthermore, the funnel plot, illustrated in **Figure 4D**, showed no indication of horizontal pleiotropy.

### 3.5 Pancreatic cancer

Our study consistently found no evidence of causal associations between T1DM and pancreatic cancer, with an OR of 1.01258 (95% CI: 0.96243-1.06533, P= 0.6294), as presented in **Table 1**. The weighted median, MR-Egger, simple mode, and weighted mode analyses produced consistent estimates, and no evidence of directional pleiotropy was detected (P>0.05). Additionally, the heterogeneity was not statistically significant (P=0.4699). The forest and scatter plots, displayed in **Figures 1E**, **2E**, respectively, further support the lack of association between T1DM and pancreatic cancer. The sensitivity analysis, presented in **Figure 3E**, revealed that no individual SNP caused the MR estimates to deviate. Furthermore, the funnel plot, illustrated in **Figure 4E**, showed no indication of horizontal pleiotropy.

### 3.6 Colorectal cancer

Our findings revealed a causal association between T1DM and colorectal cancer, with an OR of 1.000 (95% CI: 1.00045-1.0012, P<0.001), as displayed in **Table 1**. The weighted median, MR-Egger and weighted mode analyses produced consistent estimates except for simple mode, and directional pleiotropy was detected (P= 0.0134). Additionally, the heterogeneity was not statistically significant (P= 0.4699). **Figures 1F**, **2F** depict the forest and scatter plots, respectively, regarding the relationship between T1DM and colorectal cancer, indicating similar findings. As demonstrated in **Figure 3F**, the sensitivity analysis revealed that no individual SNP caused the MR estimates to deviate. Furthermore, **Figure 4F** illustrating the funnel plot, demonstrated no indication of horizontal pleiotropy.

## 4 Discussion

Our study utilised MR methods based on GWAS summary datasets to screen for possible causal associations between T1DM and six site-specific digestive cancers (the most prevalent digestive cancer types (12). We found that T1DM was causally associated with an increased risk of colorectal cancer. However, we did not observe any causal effect of T1DM on oesophageal cancer, stomach cancer, hepatocellular carcinoma, biliary tract cancer, or pancreatic cancer.

Previous observational epidemiological studies, including case-control and cohort studies, have reported inconsistent findings regarding the association between T1DM and cancer risk (10, 26). However, these studies have several limitations. Firstly, there is a possibility of misclassifying T2DM as T1DM, which could lead to an overestimation of the association between T1DM and cancer risk (11). Additionally, the criteria for defining T1DM varied across the studies. For example, Hassan et al. only mentioned insulin-dependent or non-insulin-dependent diabetes mellitus without specifying how they defined them (27), while Valent et al. defined T1DM as insulin-treated diabetes (28). Hsu et al. used the International Classification of Disease ninth version, Clinical Modification (ICD-9-CM) codes to define T1DM (29). Furthermore, most studies defined patients with T1DM as those who were 30 years old or younger, or those who were diagnosed with diabetes before the age of 30 or 45 years (11). Several studies have yielded inconsistent findings, with some early research failing to reveal significant associations between T1DM and certain types of cancer (30). For instance, large UK cohort studies indicated no increased risk or mortality from urinary bladder cancer in T1DM or T2DM patients (31, 32). However, a Netherlands Cohort Study and a Swedish study suggested a positive association between T2DM, and possibly T1DM, with the risk of invasive bladder cancer (33). Some studies show no significant link between T1DM and breast cancer risk in women, and UK and US cohort studies do not report a general increase in all-cause cancer mortality among T1DM patients; however, there are observed variations in cancer risk related to country and the duration of T1DM (34, 35). Upon examining the causal link, we identified variability among subjects. Further analysis with ebi-a-GCST90014023 (22) data did not confirm consistent findings (**Supplementary Table 3**). Caution is advised when interpreting Mendelian study outcomes, as results from different datasets can diverge or contradict each other. Finally, confounding factors such as tobacco consumption, alcohol intake, obesity, physical activity, family history of cancer, and socioeconomic status were not adjusted for in most of the included studies, which may have affected the association between T1DM and cancer risk. Therefore, conducting new research that excludes confounding factors and is based on clear definitions and patient classifications for T1DM can help identify the specific role of T1DM in the prevention and development of digestive cancers.

Likewise, our findings of the study suggest that T1DM is not a significant risk factor for oesophageal cancer, stomach cancer, hepatocellular carcinoma, biliary tract cancer, or pancreatic cancer in European populations. A cohort consisted of 23,473 UK patients with insulin-treated diabetes were followed for an average of 30 years for cancer incidence and mortality compared with general population rates showed that patients with T1DM had significantly raised risks only for ovarian and vulval cancers, with the greatest risk when diabetes was diagnosed at ages 10-14 (36). Currently, there is a relative lack of research on the potential association between T1DM and oesophagus cancer, biliary tract cancer, or pancreatic cancer. Further research is needed to fully understand the potential relationships between T1DM and these types of cancers, particularly in other populations and geographic regions.

Some previous studies have suggested that T1DM may be associated with an increased risk of stomach cancer. Zendehdel K et al. used a population-based cohort in Sweden to investigate the association between T1DM and stomach cancer risk and showed that patients with T1DM had a significantly increased risk of stomach cancer compared to the general population (37). However, these results should be interpreted with caution due to chance findings, misclassification, and several potential confounding factors. Some scholars attempted to explain this association from the perspectives of several possible mechanisms. Firstly, the long-term use of insulin to treat diabetic patients has been linked to an increase in body weight and abdominal fat deposit, which are both associated with an increased risk of stomach cancer according to a meta-analysis of cohort studies (38). Additionally, the increased risk of stomach cancer among patients with T1DM may be linked to a high prevalence of helicobacter pylori infection in those patients or a high incidence of pernicious anaemia, which is closely related to a high risk of stomach cancer because parietal cell antibodies are more frequent in patients with T1DM compared to the general population (39, 40).

The causal effects of T1DM on the risk of liver cancer remain controversial. While our study found no significant association between T1DM and liver cancer, some previous studies have suggested that T1DM may be associated with an increased risk of liver cancer (41). Possible biological mechanisms for this increased risk include alterations in hepatocellular activity, possibly mitosis related to metabolic changes in patients with diabetes, and steatohepatitis related to obesity and fibrotic confirm whether T1DM can promote liver cancer in humans and to identify the mechanisms by which T1DM exerts its effects (42).

T1DM was associated with an increased risk of colorectal cancer in our study. However, the OR value is close to 1, which suggests that T1DM may be just one of many causes of colorectal cancer. Nevertheless, since diabetes is a modifiable risk factor, its impact can be managed through interventions in daily life. Research on the relevant mechanisms conducted by Bellier J et al. aimed to investigate the link between methylglyoxal (MGO), a by-product of glycolysis, and resistance to cetuximab anti-epidermal growth factor receptor (anti-EGFR) antibodies in colorectal cancer (43). The results showed that MGO promotes tumor growth and metastasis and induces AKT activation through phosphatidylinositol 3-kinase (PI3K)/mammalian target of rapamycin 2 (mTORC2) and Hsp27 regulation, suggesting that MGO is a potential target to tackle EGFR-targeted therapy resistance in colorectal cancer. Yamagishi S et al. discussed the potential molecular link between diabetes and colorectal cancer, proposing several ways to test the hypothesis that advanced glycation end products (AGE) could explain the molecular link between diabetes and colorectal cancer (44). Oxidative stress stands out as a crucial mediator in the intricate interplay between cancer and diabetes. Reactive oxygen species (ROS), which are byproducts of this stress, are capable of modulating gene expression and pivotal pathways that are fundamental to the genesis of cancer (45). These ROS also have a hand in modulating cell proliferation and apoptosis by activating NF-κB pathways, which are frequently hyperactive in various cancers, notably colorectal (46). Furthermore, hyperinsulinemia has been associated with an increased risk of diverse cancers, encompassing the endometrium, ovary, breast, colon, pancreas, and kidney (47). The involvement of insulin and its receptor in cancer development is underscored by the fact that elevated insulin levels can augment IGF-1 production (48), a factor linked to an increased risk of specific cancers. Both IGF-1 and IGF-2 have demonstrated the ability to stimulate cancer cell proliferation and metastasis (49). The activation of the PI3K/Akt/mTOR signalling pathway by insulin and IGFs is recognised for its role in propelling cancer progression (50).

Our study has several notable strengths. Firstly, we utilised a random grouping of participants based on genotype, similar to the procedure of a randomised controlled trial, which allowed us to examine causal relationships. Secondly, we employed a MR study design, which avoids confounding biases and reverse causation commonly observed in traditional observational studies, enabling us to analyse a putative causal association between T1DM and digestive cancer. However, some limitations should be acknowledged. Firstly, genetic liability may only account for a limited proportion of the variability across individuals. Secondly, our data source primarily comprised European populations, making it challenging to generalise our results to other populations worldwide. Thirdly, the potential biological mechanism between T1DM and colorectal cancer should be further investigated with using next generation sequencing (NGS) data, such as proteomics, transcriptomics, etc. Despite these limitations, our study provides valuable insights into the causal relationship between T1DM and digestive cancer risk, which may be useful for clinicians and researchers in developing preventive strategies and interventions to mitigate the impact of this disease.

In conclusion, our study found an association between T1DM and an increased risk of colorectal cancer. However, we did not find clear evidence for a causal role of T1DM in the risk of oesophageal cancer, stomach cancer, hepatocellular carcinoma, biliary tract cancer, or pancreatic cancer. This suggests that previous associations may be confounded by potential biases or due to reverse causation.

## Data Availability

The data presented in the study are deposited in the GWAS repository (https://gwas.mrcieu.ac.uk/).
