# Supplementary material for: Casual effects of type 1 diabetes mellitus on site-specific digestive cancers: a Mendelian randomisation analysis
Source: Front Endocrinol (Lausanne). 2024 Sep 5;15:1407329. doi: 10.3389/fendo.2024.1407329 (PMC11410686; doi:10.3389/fendo.2024.1407329)
Supplement: Supplementary file 1 [file Table1.docx]

Supplementary Material

Casual Effects of Type 1 Diabetes Mellitus on Site-specific Digestive Cancer: A Mendelian Randomization Analysis

## Supplementary Tables

**Supplementary Table 1.** Baseline data of type 1 diabetes mellitus and six site-specific digestive cancers.

| **Trait** | **Year** | **Author** | **Population** | **Sample Size** | **n case** | **n control** | **n SNP** |
| --- | --- | --- | --- | --- | --- | --- | --- |
| Type 1 diabetes | 2020 | Forgetta Vincenzo | European | 24,840 | 9,266 | 15,574 | 12,783,129 |
| Oesophageal cancer | 2021 | Burrows | European | 372,756 | 740 | 372,016 | 8,970,465 |
| Malignant neoplasm of stomach | 2021 | Not available | European | 218,792 | 633 | 218,159 | 16,380,466 |
| Liver cell carcinoma | 2021 | Burrows | European | 372,184 | 168 | 372,016 | 6,304,034 |
| Malignant neoplasm of other and unspecified parts of biliary tract | 2021 | Not available | European | 218,792 | 109 | 218,683 | 16,380,466 |
| Pancreatic cancer | 2021 | Sakaue Saori | European | 476,245 | 1,196 | 475,049 | 24,195,229 |
| Colorectal cancer | 2021 | Burrows | European | 377,673 | 5,657 | 372,016 | 11,738,639 |

SNP: single nucleotide polymorphism.

**Supplementary Table 2**. A total of 35 single nucleotide polymorphisms were selected.

| **SNP** | **Chromosome** | **Effect allele** | **Non-effect allele** | **Beta** | **Standard error** |
| --- | --- | --- | --- | --- | --- |
| rs10911399 | 1 | G | A | -0.3707 | 0.064 |
| rs2269247 | 1 | T | C | 0.1709 | 0.0295 |
| rs1869449 | 2 | A | G | 0.1769 | 0.0269 |
| rs10865468 | 2 | C | G | -0.1624 | 0.0277 |
| rs11571297 | 2 | C | T | -0.1964 | 0.0237 |
| rs10183097 | 2 | C | T | 0.2053 | 0.0322 |
| rs192324744 | 2 | G | T | 0.562 | 0.0875 |
| rs6719660 | 2 | G | A | 0.2918 | 0.0524 |
| rs2111485 | 2 | G | A | 0.1577 | 0.0248 |
| rs17863786 | 2 | G | A | 0.4144 | 0.0628 |
| rs1027769 | 3 | T | G | -0.9962 | 0.1588 |
| rs62410259 | 4 | A | G | -0.3796 | 0.0533 |
| rs34954 | 5 | A | C | -0.4912 | 0.0863 |
| rs13182737 | 5 | A | G | 0.1465 | 0.0259 |
| rs9273363 | 6 | A | C | 1.2786 | 0.0334 |
| rs9296062 | 6 | C | G | 0.6913 | 0.054 |
| rs185774696 | 6 | T | C | 0.6489 | 0.0418 |
| rs206763 | 6 | A | G | 0.6792 | 0.0779 |
| rs2144013 | 6 | G | A | 0.2234 | 0.0317 |
| rs34296259 | 9 | A | T | 0.6637 | 0.1171 |
| rs10760335 | 9 | G | A | 0.1357 | 0.0243 |
| rs77523242 | 10 | C | T | -0.3705 | 0.0635 |
| rs12722495 | 10 | C | T | -0.3145 | 0.0408 |
| rs10830227 | 11 | A | G | 0.1582 | 0.0233 |
| rs79075295 | 11 | A | G | -0.4192 | 0.0621 |
| rs1131017 | 12 | G | C | -0.2461 | 0.0238 |
| rs59680223 | 12 | T | C | 0.6421 | 0.1032 |
| rs2071647 | 12 | A | T | 0.1526 | 0.0258 |
| rs201417739 | 14 | C | A | -0.416 | 0.0663 |
| rs55996894 | 14 | C | G | -0.1785 | 0.0323 |
| rs17125653 | 14 | A | T | 0.2355 | 0.0402 |
| rs4566101 | 15 | C | T | 0.1755 | 0.0255 |
| rs741172 | 16 | T | C | -0.2034 | 0.0258 |
| rs231971 | 16 | G | A | 0.2411 | 0.0399 |
| rs202520 | 20 | G | A | -0.1573 | 0.0256 |

SNP: single-nucleotide polymorphisms.

**Supplementary Table 3**. Mendelian randomization estimates for the effects of genetically determined T1DM on six site-specific digestive cancers based on ebi-a-GCST90014023 datas.

| **Site-specific digestive cancers** | **Method** | **SNPs** | **OR** | **(95% CI)** | **P-value** |
| --- | --- | --- | --- | --- | --- |
| Oesophageal cancer | Inverse variance weighted | 124 | 1.00002 | 0.99976-1.00028 | 0.8642 |
|  | MR Egger | 124 | 0.99996 | 0.9997-1.00023 | 0.8254 |
|  | Weighted median | 124 | 1.00001 | 0.99985-1.00017 | 0.8762 |
|  | Simple mode | 124 | 0.99988 | 0.99929-1.00046 | 0.6909 |
|  | Weighted mode | 124 | 1.00001 | 0.99975-1.00026 | 0.9335 |
| Stomach cancer | Inverse variance weighted | 127 | 1.0608 | 0.92313-1.219 | 0.4068 |
|  | MR Egger | 127 | 1.05696 | 0.90488-1.23459 | 0.4845 |
|  | Weighted median | 127 | 1.05981 | 0.97198-1.15558 | 0.1881 |
|  | Simple mode | 127 | 0.93933 | 0.65645-1.34409 | 0.7326 |
|  | Weighted mode | 127 | 1.09875 | 0.9682-1.24689 | 0.1469 |
| Hepatocellular carcinoma | Inverse variance weighted | 100 | 0.99992 | 0.99977-1.00007 | 0.3189 |
|  | MR Egger | 100 | 0.99992 | 0.99979-1.00006 | 0.2786 |
|  | Weighted median | 100 | 0.99999 | 0.9999-1.00008 | 0.8837 |
|  | Simple mode | 100 | 1.00024 | 0.99993-1.00055 | 0.1244 |
|  | Weighted mode | 100 | 0.99996 | 0.99983-1.00009 | 0.6285 |
| Biliary tract cancer | Inverse variance weighted | 127 | 0.96999 | 0.68879-1.366 | 0.8618 |
|  | MR Egger | 127 | 1.15522 | 0.81668-1.63408 | 0.4147 |
|  | Weighted median | 127 | 1.07055 | 0.86604-1.32337 | 0.5284 |
|  | Simple mode | 127 | 1.01356 | 0.47177-2.17752 | 0.9725 |
|  | Weighted mode | 127 | 1.12055 | 0.79334-1.58271 | 0.5194 |
| Pancreatic cancer | Inverse variance weighted | 138 | 0.9799 | 0.89728-1.07013 | 0.6522 |
|  | MR Egger | 138 | 1.01693 | 0.92347-1.11985 | 0.7328 |
|  | Weighted median | 138 | 1.00587 | 0.953-1.06166 | 0.8316 |
|  | Simple mode | 138 | 1.00499 | 0.81345-1.24162 | 0.9632 |
|  | Weighted mode | 138 | 0.99113 | 0.91304-1.07591 | 0.8319 |
| Colorectal cancer | Inverse variance weighted | 128 | 1.00065 | 0.99996-1.00134 | 0.0642 |
|  | MR Egger | 128 | 1.00063 | 0.99995-1.00132 | 0.0671 |
|  | Weighted median | 128 | 0.99986 | 0.99942-1.0003 | 0.5425 |
|  | Simple mode | 128 | 0.99984 | 0.99805-1.00164 | 0.8684 |
|  | Weighted mode | 128 | 1.00053 | 0.99984-1.00122 | 0.1304 |
